# Prospective evaluation of implants‐supported, tooth‐implant supported, and teeth‐supported 3‐unit posterior monolithic zirconia fixed restorations: Bite force and patient satisfaction

**DOI:** 10.1002/cre2.780

**Published:** 2023-09-13

**Authors:** Sadeq Altayyar, Walid Al‐zordk, Radwan Algabri, Eshraq Rajah, Abdulsattar Al‐baadani, Ahmed Yaseen Alqutaibi, Manal Abo Madina, Mohammed H. Ghazy

**Affiliations:** ^1^ Prosthodontic Department, Faculty of Dentistry IBB University Ibb Yemen; ^2^ Fixed Prosthodontic Department, Faculty of Dentistry Mansoura University Mansoura Egypt; ^3^ Prosthodontic Department, National University Ibb‐Branch Ibb Yemen; ^4^ Prosthodontic Department, Faculty of Dentistry Sana'a University Sana'a Yemen; ^5^ Fixed Prosthodontic Department, Faculty of Dentistry Dhamar University Dhamar Yemen; ^6^ Department of Prosthodontic and Implant Dentistry, College of Dentistry Taibah University Al Madinah Saudi Arabia; ^7^ Department of Prosthodontics, College of Dentistry Ibb University Ibb Yemen

**Keywords:** biting force, implant, monolithic zirconia, patient satisfaction

## Abstract

**Objectives:**

This study aimed to evaluate the maximum bite force (MBF) and satisfaction of patients restored with implants, combined tooth‐implants, and teeth‐supported monolithic zirconia fixed dental prostheses (FDPs).

**Materials and Methods:**

Thirty partially edentulous patients in need of three units of FDPs in their mandibular posterior region were divided into three equal groups (*n* = 10) as follows: Group‐1 patients received two implants for each at the second premolar and second molar regions, Group‐2 patients received one implant for each at the second molar region, and Group‐3 patients with missing lower first molar. All the restorations were constructed from monolithic zirconia. Patients were evaluated 1 week after placement of restorations (baseline) and then after 6, 12, and 24‐month intervals for MBF using force transducer occlusal force meter and satisfaction (function, esthetic, and overall satisfaction) using a visual analog scale.

**Results:**

The mean MBF for Group 1 was higher than Group 2 (*p* = .044) but not that of Group 3 (*p* = .923). Additionally, Group 3 displayed a higher MBF than Group 2, although this difference was not statistically significant (*p* = .096). Concerning patient satisfaction, all study groups reported high levels of satisfaction across all satisfaction elements, and no significant differences were observed between the groups.

**Conclusion:**

Within the limitations of this study, it can be concluded that Group 1 gives comparable anticipated treatment outcomes as Group 3 concerning biting force and patient satisfaction. However, Group 2 gives comparable satisfaction results with biting force value within the normal range; thus, it might be used as a treatment option in a specific situation.

## 1 INTRODUCTION

Nowadays, implant‐supported prosthodontics represents one of the treatment options in fixed prosthodontics, in which the treatment with dental implants appears to improve oral function significantly and increase patient satisfaction (Beuer et al., 2016; Lemos et al., 2022). This approach has been documented and accepted as a contemporary clinical method with expectable long‐term success for rehabilitation of partially as well as entirely edentulous patients; it could offer the possibility of long‐term stabilizing oral prostheses such as fixed dental prostheses (FDPs) and overdentures with minimum complications and thus may overwhelm some of the functional limits of conventional dentures (Alqutaibi et al., 2021; Hatim, 2014; Muddugangadhar et al., 2015; Pozzi et al., 2015).

The combination of support from a connection of implants to natural teeth has been considered in many studies but remains controversial (Borg et al., 2016; La Monaca et al., 2021; Ramoglu et al., 2013; Tsaousoglou et al., 2017). Nevertheless, many clinical situations may necessitate an implant's connection to natural teeth to support FDPs, making the placement of other implants and bone augmentation impossible. These situations as in the patients with bruxism in which proprioception of the tooth may aid in reducing applied stresses to the implants, (Ghodsi & Rasaeipour, 2012; Spear, 2009) the systemic, local, or financial limits, (Michalakis et al., 2012) tooth with compromised periodontium, and anatomical limitations as an approximation of maxillary sinus, inadequate position of the mandibular canal and mental foramen (Gabbert et al., 2012; Michalakis et al., 2012).

One of the primary essential findings when comparing osseointegrated implants with natural teeth is the dissimilarity in the amount of potential movement when the force is applied. The connection methods between the implant and natural tooth may be rigid or nonrigid. Nonrigid connections could be in the attachment (either precision or semiprecision), telescopic restorations, and intermobile elements (Ghodsi & Rasaeipour, 2012; Shenoy et al., 2013).

Over the previous decade, there have been significant technological advances in the field of dental ceramics and increasing requests for metal‐free dental prostheses mostly because of improvements in strength, clinical performance, longevity, cost, esthetics, and biosafety that have made all‐ceramic restorations more popular and more predictable (Pollington & van Noort, 2009). Furthermore, all‐ceramic restorations have been recently constructed using computer‐aided design/computer‐assisted manufacturing (CAD/CAM), allowing for control of time and fee to a proper degree (Sulaiman et al., 2015).

After the clinical success of tooth‐retained zirconia‐based FDPs, the clinicians extended the application of zirconia ceramics for implant‐supported crowns as well as FDP, in which numerous studies have presented that zirconia has adequate strength to function as a framework for posterior FPDs (Alqutaibi et al., 2022; Sorrentino et al., 2012). Although the veneering ceramic chipping and fracture was the most common complication, (Al‐Amleh et al., 2010; Esquivel‐Upshaw et al., 2014; Heintze & Rousson, 2010; Ioannidis & Bindl, 2016; Konstantinidis et al., 2015; Sorrentino et al., 2012; Sulaiman et al., 2015) the idea of constructing a prosthesis made of complete zirconia (full contour) material was approached by eliminating the veneering ceramic and depending on stains and glazes layering or a glaze spray technique for achievement of esthetic appearance (Ioannidis & Bindl, 2016; Kern et al., 2012; Stawarczyk et al., 2013).

Maximum bite force (MBF) measurement can be used as a parameter indicating the functional condition of the masticatory system, (Koc et al., 2010) assessment of the prosthetic treatment and evaluation of oral function concerning occlusal factors, dentition, dental prostheses, implant treatment, orthognathic surgery, oral surgery, temporomandibular disorders, and neuromuscular disease. This measurement can be recorded either by directly utilizing an appropriate transducer which is a helpful method for assessing the maximum voluntary bite force, strain gauges, piezoelectric sensors, and pressure sheets, or by indirect evaluation using electromyography (Van Der Bilt et al., 2008; Röhrle et al., 2018; Tripathi et al., 2014).

For determining the treatment success of implant‐supported prostheses and obtaining the patient's well‐being, there are many different parameters for this issue; one of the most common parameters is based on patient satisfaction (Gurgel et al., 2015). The use of satisfaction rating as an outcome in comparison to intraoral restorations is commonly used (Cheng et al., 2012). Different methods measure patient satisfaction; simple verbal rating scale (e.g., Likert scale), numerical rating scale, and visual analog scale (VAS) (Brokelman et al., 2012; Hjermstad et al., 2011).

Several research studies concentrated on the success or failure of implants according to biological standards, while few studies rendered the prosthetic results based on the patient's perception (Beuer et al., 2016; Lekholm et al., 2006). In addition, other studies evaluated and compared the satisfaction and oral health‐related quality of life of patients wearing removable prostheses and implant‐supported overdentures (ELsyad et al., 2019; Goiato et al., 2015) which exposes the lack of data about patients wearing implants‐supported, combined tooth‐implant and teeth supported fixed partial dentures. Furthermore, no clinical data supports full‐contour zirconia FDPs on implants.

Thus, this study aimed to evaluate the biting force and satisfaction of patients restored by implant‐supported, tooth‐implant supported and compare them with teeth‐supported fixed monolithic zirconia ceramic restorations. The null hypothesis to be tested was that there is no difference between implant‐supported, tooth‐implant supported, or teeth‐supported fixed monolithic zirconia restorations regarding biting force and patient satisfaction.

## 2 MATERIALS AND METHODS

The present study recruited 30 patients (four males and 26 females) from the Fixed Prosthodontics Department, Faculty of Dentistry, Mansoura University, Egypt, over 3 years, from May 2016 to May 2019. The sample was composed of individuals who required three units of FDPs in the lower posterior region, with a mean age of 35.6 years (range: 26–50 years). Before participation, all patients provided written informed consent, and the university's ethical committee approved the study protocol.

The required sample size was calculated using a single population mean formula of 0.05 with a power of 80% and a confidence level of 95%. Thirty patients (10 patients per group) were calculated to be adequate to detect the effect size of MBF.

### 2.1 Inclusion and exclusion criteria

In the present study, patient selection was based on specific criteria for each group. Group 1 included patients with unilateral or bilateral free‐end saddle (Kennedy classes I or II) in the lower jaw, with the terminus abutment being the first premolar. Group 2 included patients with the terminus abutment being the second premolar. Group 3 consisted of patients with a missing mandibular first molar. The opposing jaw was completely dentate or had simple operative restorations for all study groups. The exclusion criteria were patients with bruxism, clenching habits, or temporomandibular joint disorders.

### 2.2 Patient grouping and procedures

Patients were distributed into three equal groups according to the abutment support. Group‐1 patients were recruited for implant‐supported FDPs, Group‐2 patients were recruited for combined tooth‐implant supported FDPs, and Group‐3 patients were recruited for tooth‐supported FDPs.

After the patient's history was reviewed, intra and extra‐oral examination and cone‐beam computed tomography were obtained, Group‐1 received 20 implants (Neo Biotech Co., Ltd.) that were placed at regions of mandibular first premolar (10 implants) and mandibular second molar (10 implants). In contrast, for Group 2, 10 implants were placed at the regions of the second molar.

A healing period of 3–4 months was allowed. A digital peri‐apical radiograph for osseointegration assessment was performed, and a tissue punch was used for eradicating soft tissue and exposing the cover screw using a guide splint; the gingival healing former was carefully screwed for 2 weeks.

The natural tooth abutments (mandibular second premolar) in Group 2 and mandibular second (premolar and molar) in Group 3 were prepared in the same manner according to the manufacturer's recommendation (occlusal reduction of 1.5–2 mm, axial reduction of 1–1.5 mm, and deep chamfer preparation). Marginal preparations were located supragingivally.

In Groups 1 and 2, an open tray impression technique was taken utilizing custom trays from acrylic resin (ACrostone; Dental and Medical Suppliers El Hegaz St.) using double‐mix one‐step technique additional silicon impression material (Betasil, heavy‐body and light‐body; Muller‐Omicron Gmbh & Co KG Germany), while in Group 3, the double‐mix one‐step conventional impression technique was took using the same materials.

The implant abutments (Neo Biotech Co., Ltd.) were negotiated into the implant analog in the master cast and assessed for parallelism with the premolar and were prepared using a milling machine (Wieland Dental A Company of Ivoclar Vivadent Group).

Temporary FDPs were fabricated from CAD/CAM composite block (unfilled poly(methyl methacrylate)‐based high‐density polymer block, Telio CAD; Ivoclar Viva Dent) and cemented with noneugenol temporary cement (Temp Bond NE; Kerr) for all groups.

Full anatomic monolithic zirconia FDPs were designed using the software package Dental Designer™ (Wieland Dental GmbH). The minimal connector section area was 9 mm^2^, and the minimal retainer thickness was 1–1.5 mm, as recommended by the manufacturer. The scanned data were enlarged by 20%–25% to compensate for sintering shrinkage and the restorations, then milled from presintered zirconia blank (Zolid High Translucent Pre‐shade Amanngirrbach GmbH). All restorations were checked intraorally for their marginal accuracy with dental explorer, the internal wall fitness with a silicone disclosing agent (Fit Checker; GC America Inc.), the contact area and occlusion using an articulating ribbon (TRU SILK Articulating Ribbon MDS Product Inc.).

**Figure 1:**
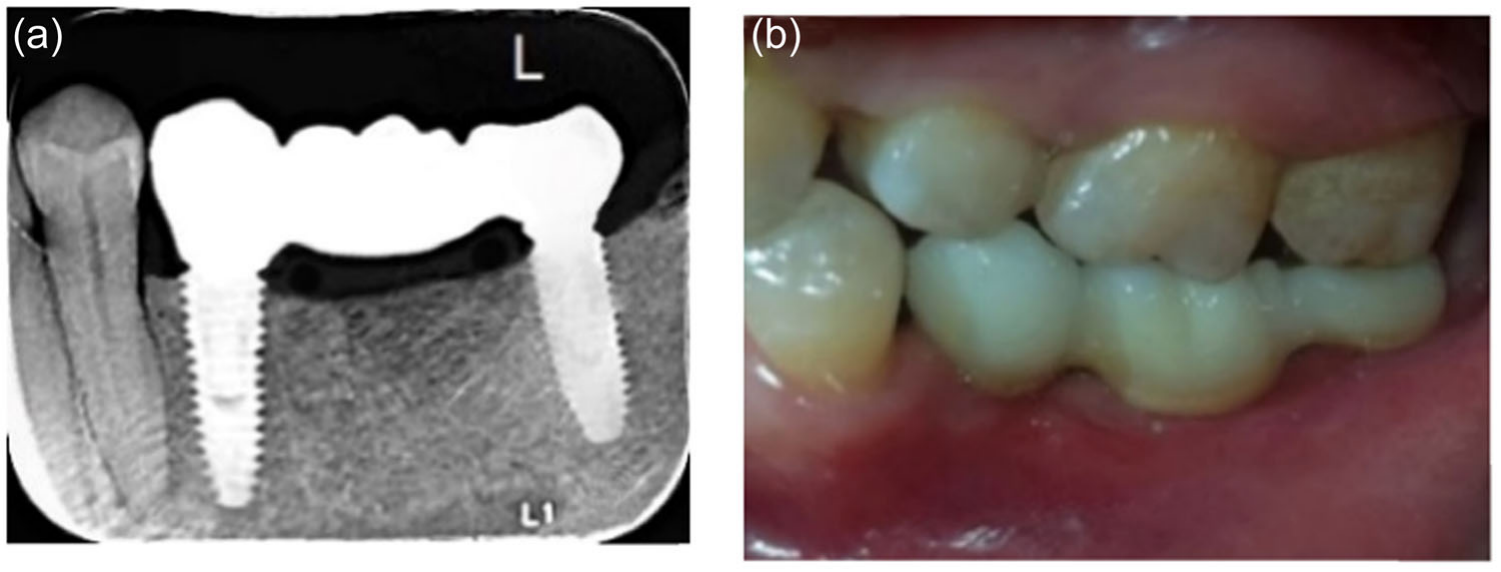
Implant‐supported FPDs (Group 1). (a) Periapical radiograph and (b) clinical photograph. FDPs, fixed dental prostheses.

**Figure 2:**
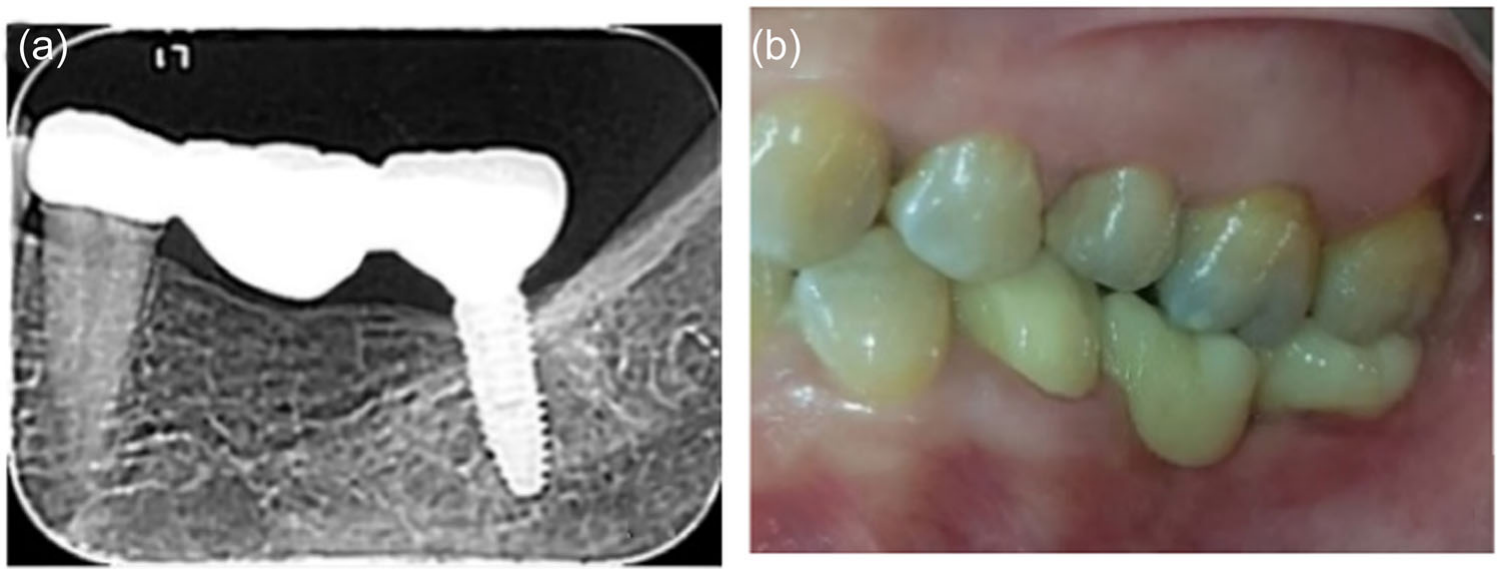
Implant‐tooth supported FPDs (Group 2). (a) Periapical radiograph and (b) clinical photograph. FDPs, fixed dental prostheses.

**Figure 3:**
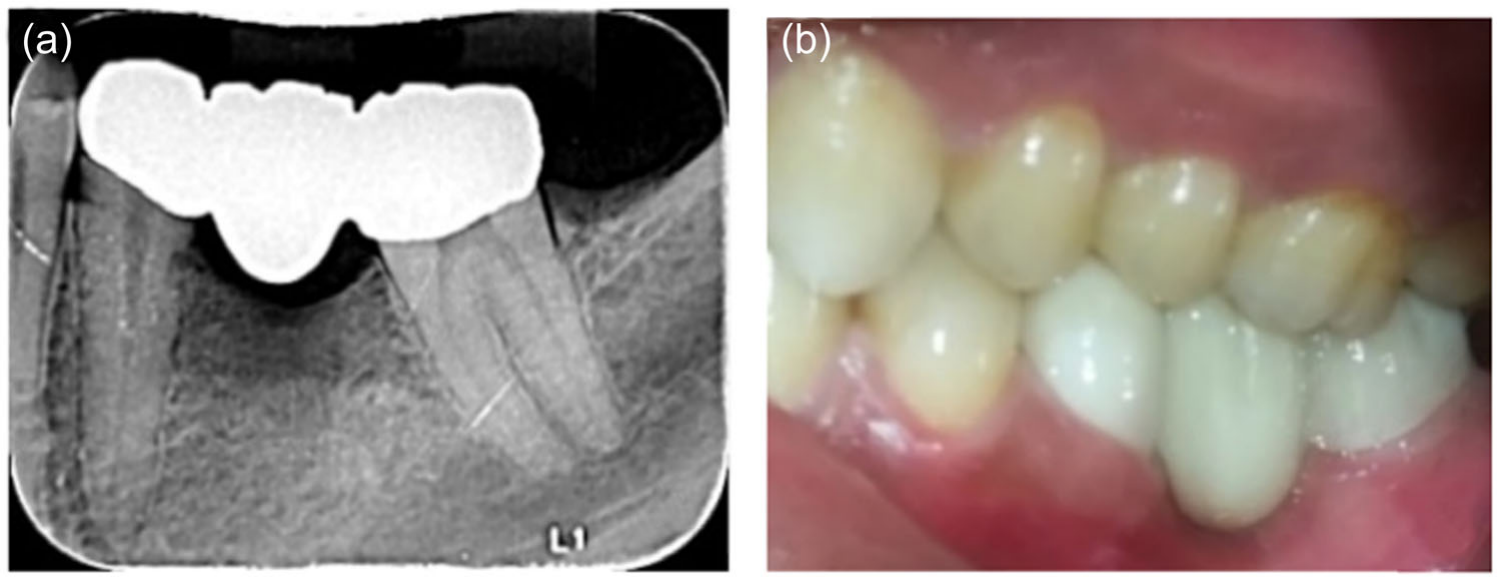
Tooth‐supported FPDs (Group 3). (a) Periapical radiograph and (b) clinical photograph. FDPs, fixed dental prostheses.

**Figure 4:**
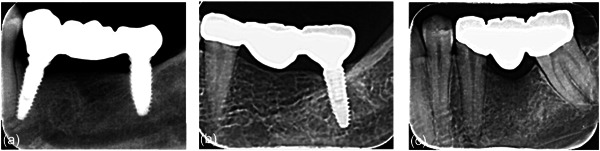
Radiographs from the last evaluation period (a) Group 1, (b) Group 2, and (c) Group 3.

All FDPs were cemented with glass ionomer cement (Medicem Glass Ionomer Luting Cement; PROMEDICA Dental Material GmbH). (Figures 1, 2, 3). All patients were examined 1 week after insertion of the restorations (baseline) and then after 6, 12, and 24‐month intervals (Figure 4).

### 2.3 Maximal biting force assessment

**Figure 5:**
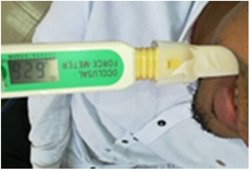
Bite force measurement using occlusal force meter (GM10; Nagano Keiki).

Bite force was measured at the occlusal surface of the first mandibular artificial FDPs on the restored side (Abu Alhaija et al., 2010; Al‐Zarea, 2015) using a force transducer occlusal force meter (GM10; Nagano Keiki) that comprised of a digital hydraulic pressure gauge and a vinyl biting component encased with plastic envelop. The pressure gauge exposed the bite force values in newtons on its digital screen. All patients were in an upright position in the dental chair while measuring. Subsequently, every patient was asked to bite as much as possible on the bite gauge. The process was repeated three times for each patient at 45‐s intervals in each visit, and the highest value was recorded (Figure 5).

### 2.4 Patient satisfaction assessment

A VAS ranging from 0 to 10 (horizontal line of 100‐mm long) was used. Two descriptors are present at the beginning and the end of the line representing the satisfaction extremes (i.e., 0 represented the lowest or no satisfaction while 10 represented the highest or extreme satisfaction). The patient valued his satisfaction by writing a vertical mark on the 100‐mm line in which the numbers selected by patients corresponded to their degree of satisfaction with the outcomes. Patients assigned scores (1 ≥ 50, 2 ≥ 60, 3 ≥ 70, 4 ≥ 80, and 5 ≥ 90) to the following three aspects: function, esthetics, and overall satisfaction (Andrade de Lima et al., 2012; da Cunha et al., 2015).

### 2.5 Data processing/statistical analysis

The data were collected, tabulated, and then analyzed using SPSS software (version 23 SPSS Inc.). The tested variables were normally distributed using the Kolmogorov–Smirnov test for biting force. The mean value was compared by (analysis of variance [ANOVA]) one‐way variation analysis test to compare between the study groups. For patient satisfaction, data were summarized as mean ranks, and the Kruskal–Wallis test compared ranks' values.

## 3 RESULTS

A total of 30 FDPs (3‐unit full anatomic zirconia bridge) were placed in 30 participants according to the supporting substrates, all of which had complete occlusal contact with teeth in the opposite arch. Three participants were lost (one for each group) to follow‐up during the observation period (24 months).

### 3.1 Maximal biting force

One‐way ANOVA test for biting force represented a statistically significant difference between the study groups (*p* = .037). The post hoc (Tukey honestly significant difference) test was used for multiple comparisons between study groups, each one to another; it represented bite force in Group 1 was higher than in Group 2 (*p* = .044), while no significant difference with Group 3 (*p* = .923). Furthermore, the bite force of Group 3 was higher than Group 2 with no significant difference (*p* = .096) (Tables 1 and 2).

**Table 1:** Means and SD of bite force (*N*) in each study group.

Groups	Number	Mean bite force ± SD *N*
Implant–implant‐supported FDPs (Group 1)	9	415.87 ± 64.89
Tooth‐implant supported FDPs (Group 2)	9	339.32 ± 86.47
Tooth–tooth‐supported FDPs (Group 3)	9	404.46 ± 67.88
Total	27	3404.46 ± 67

Abbreviations: FDP, fixed dental prostheses; SD, standard deviation.

**Table 2:** One‐way ANOVA test for biting force between study groups.

Biting force	Sum of squares	*df*	Mean square	*F*	Sig.
Between groups	30,704.524	2	15,352.262	3.800	0.037
Within groups	96,964.813	24	4040.201		
Total	127,669.336	26			
*p* Significant at ≤.05*					

Abbreviation: ANOVA, analysis of variance.

* *p*‐Value.

### 3.2 Patient satisfaction

For patient satisfaction, the Kruskal–Wallis test represented no difference between the study groups regarding function, esthetic, and overall satisfaction during the observational period (Tables 3 and 4).

**Table 3:** Kruskal–Wallis test of patient satisfaction between study groups.

Satisfaction	Function (baseline)	Function (6 months)	Function (12 months)	Function (24 months)
*χ* ^2^	2.375	2.140	0.295	2.167
*df*	2	2	2	2
Sig.	0.305	0.343	0.863	0.338

Satisfaction	**Esthetic (Baseline)**	**Esthetic (6 months)**	**Esthetic (12 months)**	**Esthetic (24 months)**
*χ* ^2^	3.822	0.444	2.400	4.627
*df*	2	2	2	2
Sig.	0.148	0.801	0.301	0.099

Satisfaction	**Overall satisfaction (baseline)**	**Overall satisfaction (6 months)**	**Overall satisfaction (12 months)**	**Overall satisfaction (24 months)**
*χ* ^2^	2.199	4.345	2.413	0.726
*df*	2	2	2	2
Sig.	0.333	0.114	0.299	0.695
*p* Significant at ≤.05*				

* *p*‐Value.

**Table 4:** Tests of normality of patient satisfaction for study groups.

Groups of the study	Shapiro–Wilk
	Sig.
Function baseline	
Group 1: Implant–implant	0.215
Group 2: Tooth‐implant	0.024
Group 3: Tooth–tooth	0.340
Function 6 months	
Group 1: Implant–implant	0.001
Group 2: Tooth‐implant	0.055
Group 3: Tooth–tooth	0.024
Function 12 months	
Group 1: Implant–implant	0.000
Group 2: Tooth‐implant	0.000
Group 3: Tooth–tooth	0.000
Function 24 months	
Group 1: Implant–implant	0.000
Group 2: Tooth‐implant	0.000
Esthetic baseline	
Group 1: Implant–implant	0.001
Group 2: Tooth‐implant	0.024
Group 3: Tooth–tooth	0.000
Esthetic 6 months	
Group 1: Implant–implant	0.001
Group 2: Tooth‐implant	0.000
Group 3: Tooth–tooth	0.000
Esthetic 12 months	
Group 1: Implant–implant	0.000
Group 2: Tooth‐implant	0.001
Group 3: Tooth–tooth	0.000
Esthetic 24 months	
Group 1: Implant–implant	0.000
Group 2: Tooth‐implant	0.001
Group 3: Tooth–tooth	0.000
Overall satisfaction baseline	
Group 1: Implant–implant	0.028
Group 2: Tooth‐implant	0.008
Group 3: Tooth–tooth	0.000
Overall satisfaction 6 months	
Group 1: Implant–implant	0.028
Group 2: Tooth‐implant	0.000
Group 3: Tooth–tooth	0.000
Overall satisfaction 12 months	
Group 1: Implant–implant	0.000
Group 2: Tooth‐implant	0.028
Group 3: Tooth–tooth	0.000
Overall satisfaction 24 months	
Group 1: Implant–implant	0.000
Group 2: Tooth‐implant	0.002
Group 3: Tooth–tooth	0.000

## 4 DISCUSSION

The null hypothesis was rejected regarding MBF as there were differences observed between the study groups, while it was accepted regarding patient satisfaction as there were no differences observed between the groups.

The results from this study demonstrated that full anatomic monolithic zirconia FDPs were used for evading the problem of ceramic veneer chipping that commonly occurs on zirconia framework restoration and gaining the benefits of zirconia's strength, as reported by (Ioannidis & Bindl, 2016), and (Kern et al., 2012) and to reduce the amount of wear that occurs in the antagonist natural teeth as reported by (Stawarczyk et al., 2013).

In this study, the restorations were placed as 3 unit FDPs on different substrates (i.e., implants, combined tooth implants, and teeth) through which the applications of full anatomic zirconia have been immense in the dental arena, involving single and multiple unit restorations, abutments, and full arch implant retained restorations as stated by Anusavice (2013).

Temporary FDPs were used for the maintenance of occlusal relation and further verification. The final restorations with rigid connections were used to avoid dental intrusion and decrease the mechanical failure rate (Cordaro et al., 2005; Ghodsi & Rasaeipour, 2012; Nickenig et al., 2006; Shenoy et al., 2013).

Bite force might be an essential factor in implant selection and planning of prosthetic cases, particularly in patients who can deliver very high occlusal force (risk for overload developing and consequent implant failure), so the magnitude of the patient's bite force may be an issue for a long‐term successful outcome (Flanagan, 2017).

In this study, we use the direct method for bite force measurement dependent on earlier studies which have proved that the direct method of bite force measuring is more convenient and accurate when compared to indirect evaluation by employing an electromyography test for the masseter and temporalis muscles (Gurgel et al., 2015; Tripathi et al., 2014). Force transducer occlusal force meter was used in this study due to its accuracy and precision, which had been formerly confirmed, as well as its ease of use; no need for any specific mounting, it has a smaller thickness (8.6‐mm‐thick bite part), with no interference with the tongue in addition to the simplest of disinfection by changing the disposable plastic cover (Abu Alhaija et al., 2010; Al‐Zarea, 2015; Serra & Manns, 2013). MBF measured at the restored side for all study groups was within the wide range (213–1500 N) recorded previously (Abu Alhaija et al., 2010; Al‐Omiri et al., 2014; Al‐Zarea, 2015; Calderon et al., 2006; Miyaura et al., 2000).

A VAS was used in the current study to assess patient satisfaction as it appears to be one of the most common and reliable methods (Beuer et al., 2009; Brokelman et al., 2012; da Cunha et al., 2015; Håff et al., 2015; Sherif et al., 2011).

The present study revealed that the highest biting force measurement was for Group 1: implants‐supported FDPs (415.87 ± 64.89 N) followed by Group 3: tooth‐supported FDPs, (404.46 ± 67.88 N), then Group 2: tooth‐implant supported FDPs (339.3 ± 86.47 N); this could be explained by the dissimilarity in the amount of potential movement when the force is applied, as the natural teeth are attached to the alveolar bone using periodontal ligament fibers, whereas osseointegrated implant is rigidly anchored to the bone (Shenoy et al., 2013).

Furthermore, the naturally healthy tooth can move 200 µm in response to a 0.1 N force, while an implant can be moved 10 µm or less, (Shenoy et al., 2013) sometimes less than 0.1 µm (Ramoglu et al., 2013). Intact tooth has 8–28 µm physiological vertical movement while this movement is 0–5 µm for implant. Horizontal movements are more excessive than vertical ones. Teeth make moves 56–108 µm even with small forces like 500 g (Ramoglu et al., 2013), compared to 10–50 µm in the implant with the same force magnitude (Ghodsi & Rasaeipour, 2012), so the forces applied to the tooth are perceived by periodontal and intradental mechanoreceptors, which are only activated by the rapid stimulus (Serra & Manns, 2013).

The difference in biting force between implants‐supported (Group 1) and tooth‐supported (Group 3) compared to tooth‐implant supported FDPs (Group 2) was simple and comparable to each other, which situates the treatment by implants‐supported 3 unit bridge a favorable option; this was following (Pol et al., 2018). who systematically reviewed and stated that implant‐retained 3‐unit FPDs seem to be a reliable treatment with survival rates, complications, and patient‐reported outcome measures not considerably different from the outcomes of teeth‐retained 3‐unit FPDs.

In comparison, the MBF of Group 1 was higher than Group 2; however, several studies by Rammelsberg et al. (2013) and Mostafa et al. (2015) concerned many outcomes other than MBF. They stated that “the occurrence of short‐term failure of implant‐retained and tooth‐implant‐retained FPDs was minimal, the complications for tooth‐implant retained FDPs was not higher than for implant‐retained FPDs as well as tooth‐implant retained restorations provides similarly expectable treatment outcomes as the implant‐retained restorations regarding implant survival and marginal bone loss.”

MBF of Group 3 was higher than Group 2; this may be interpreted by the protective value of proprioception provided by the tooth connected to the implant and more bone reaction as the FDPs connected to both the implant and teeth (Ghodsi & Rasaeipour, 2012; Greenstein et al., 2009; Mostafa et al., 2015; Ramoglu et al., 2013; Shenoy et al., 2013)., in addition to the activation of protective sensory mechanism from the mechanoreceptors that exist in the periodontal ligament around opposite natural teeth, might play a possible cause for comparable MBF for implant and tooth supports as well as the evolutionary progress of peri‐implant proprioception via coupling of existing nerve fibers in the bone to the implant surface (Akca et al., 2006; Wada et al., 2001) and so some patients in Group 2 were afraid of fracture (either tooth or restoration) from maximal biting force exertion due their heterogeneous substrate and reporting some degree of pain on the opposing dentition during the MBF recording. Furthermore, it could be interpreted that when the implant is connected to the teeth, unbalanced force distribution between tooth and implant will occur as the implant always carries a significant part of loading (Cheng et al., 2017).

MBF of Group 2 (339.3 ± 86.47 N) was in the range that was recorded by Michalakis et al. (2012) (264–336 N). The biting force value of Group 3 was in the range of natural teeth bite tested by (Sano & Shiga, 2021) and Ferrario et al. (2004); instead, it was of lower value than that conveyed by (Al‐Zarea, 2015) and Abu Alhaija et al. (2010). This can be interpreted by the difference of races which may have different biting forces attributed to different eating habits, body features, lifestyles, physical and psychological states, and different facial morphology. Another issue, for instance, the use of different devices with different thicknesses of biting components, methods, and control of measuring processes could also play a role in the magnitude of MBF observed in various studies (Abu Alhaija et al., 2010; Al‐Zarea, 2015; Anusavice, 2013).

Regarding patient satisfaction, all study groups exposed high satisfaction with no difference between satisfaction parameters, and this could be illustrated by the success of their implant‐supported restorations and their social effect on the daily routine. This was in agreement with a previous study (Kumar et al., 2017), which reported overall satisfaction with restorations in addition to satisfaction with function after treatment with implants; this probably could be a result of the feeling of more advanced treatment administered without compromising the health of neighboring teeth and easier maintainability as well as implant‐supported prosthesis may feel more natural, and with Annibali et al. (2010) and Goiato et al. (2015) who stated in their studies that the patients were highly satisfied with the chewing function in which 82.7% of them were able to chew any food, 84.6% reflecting that the restorations were similar to their natural bite and considering that construction as an integral part of their mouth, regarding the outcome of implant treatment, the satisfaction was 82.7%, and 86.5% were agreeable to performed the same treatment again. In the same context, Marjan et al. (2012) revealed that the satisfaction of patients treated with implant‐supported fixed prosthesis and implant‐supported crown was 96% for chewing ability, 91% for comfort with the restorations, and 86% were satisfied with the esthetic appearance of restorations.

The limitations of this study included the following: First, the study's sample size was relatively small, which may limit the generalizability of the findings. Second, the study excluded patients with bruxism, clenching habits, and temporomandibular joint disorders, which may limit the applicability of the findings to these patient populations. Third, the study evaluated patients for 24 months after restoration placement, while this may be sufficient to assess short‐term outcomes. More extended follow‐up periods may be necessary to evaluate the long‐term success of the restorations. Finally, we only evaluated static parameters of masticatory efficiency through MBF measurement, which may not be enough to assess the dynamic nature of masticatory function fully. A more comprehensive evaluation of masticatory efficiency could include the assessment of other dynamic parameters, such as chewing, speed, occlusal contact area, and food particle size reduction.

To address these limitations, it is recommended that future studies conduct a multicenter, well‐designed clinical trial that evaluates a broader range of sequentially selected participants. This study should include a comprehensive assessment of dynamic parameters of masticatory efficiency, providing more convincing evidence and a more accurate representation of the clinical significance of the evaluated restorations.

## 5 CONCLUSIONS

Within the limitation of this study, the following conclusions were drawn from the results of the present 2‐year follow‐up clinical evaluation:

1. Patients with mandibular posterior 3‐unit FDPs supported by implants have the highest MBF compared to patients with tooth‐implant and teeth‐supported mandibular restorations.
2. Patient satisfaction is high regardless of the supporting substrates (implants, tooth implants, and teeth).
3. Connecting implants rigidly to teeth gives a comparable satisfaction result with a biting force value within the normal range; thus, it might be a treatment option; However, this should be done with caution as this is based on short‐term observation.

## AUTHOR CONTRIBUTIONS

**Sadeq Altayyar**: Conceptualization; methodology; investigation; analysis; and writing the original draft. **Walid Al‐Zordk**: Conceptualization; methodology; investigation. **Radwan Algabri**: Methodology; investigation; critically revised the manuscript. **Eshraq Rajah**: Conceptualization; methodology; investigation. **Abdulsattar Al‐baadani**: data collection; interpretation; critically revised the manuscript. **Ahmed Alqutaibi**: Critically revised the manuscript and analysis. **Manal Abo Madina**: Methodology; investigation; analysis and supervision. **Mohammed H. Ghazy**: Methodology; investigation and supervision.

## CONFLICT OF INTEREST STATEMENT

The authors declare no conflict of interest.

## ETHICS STATEMENT

The patients gave written informed consent, and the study was approved by the ethical committee of the Fixed Prosthodontics Department, Faculty of Dentistry, Mansoura University.

## Data Availability

The data that support the results of this trial are available from the corresponding author, (Radwan Algabri) when requested.
